# Heterogeneous 2D/3D photonic integrated microsystems

**DOI:** 10.1038/micronano.2016.30

**Published:** 2016-08-01

**Authors:** S. J. Ben Yoo, Binbin Guan, Ryan P. Scott

**Affiliations:** 1 Department of Electrical and Computer Engineering, University of California, Davis, CA 95616, USA

**Keywords:** photonic integrated circuits, three-dimensional photonics, ultrafast laser inscription

## Abstract

The continuing trend of exponential growth in data communications and processing are driving the need for large-scale heterogeneous integration. Similar to the trend we have observed in electronic integrated circuit development, we are witnessing a growing trend in 3D photonic integrated circuits (PICs) development in addition to that in 2D PICs. There are two main methods for fabricating 3D PICs. The first method, which utilizes ultrafast laser inscription (ULI), offers freeform shaping of waveguides in arbitrary contours and formations. The second method, which utilizes multilayer stacking and coupling of planar PICs, exploits relatively mature 2D PIC fabrication processes applied to each layer sequentially. Both the fabrication methods for 3D PICs have advantages and disadvantages such that certain applications may favor one method over the other. However, a joining of 2D PICs with 3D PICs can help develop integrated microsystems with new functionalities such as non-mechanical beam steering, space-division multiplexing (SDM), programmable arbitrary beam shaping, and photonic signal processing. We discuss examples of 3D PICs and 2D/3D integrated PICs in two applications: SDM via orbital-angular-momentum (OAM) multiplexing/demultiplexing and optical beam steering using optical phased arrays. Although a 2D PIC by itself can function as an OAM multiplexer or demultiplexer, it has limitations in supporting both polarizations. Alternatively, a 3D PIC fabricated by ULI can easily support both polarizations with low propagation loss. A combination of a 3D PIC and a 2D PIC designed and fabricated for OAM applications has successfully multiplexed and demultiplexed 15 OAM states to demonstrate polarization-diversified SDM coherent optical communications using multiple OAM states. Coherent excitation of multi-ring OAM states can allow highly scalable SDM utilizing Laguerre–Gaussian modes or linearly polarized (LP) modes. The preliminary fabrication of multi-ring OAM multiplexers and demultiplexers using the multilayer 3D PIC method and the ULI 3D PIC method has also been pursued. Large-scale (for example, 16×16 optical phased array) 3D PICs fabricated with the ULI technique have been demonstrated. Through these examples, we show that heterogeneous 2D/3D photonic integration retains the advantages of 2D PICs and 3D waveguides, which can potentially benefit many other applications.

## Introduction: Why photonics? Why heterogeneous integration? Why 3D?

The amount of information we process has been continuing its remarkable trend of exponential growth. Sustainably supporting such explosive growth requires scalable, high-yield, and cost-effective integration of microsystems. For two-dimensional electronic integrated circuits (2D EICs), Moore’s law stated more than five decades ago^1^ that the number of transistors that could be placed inexpensively on an electronic integrated circuit (EIC) doubled approximately every 2 years. As a corollary, Dennard’s law^2^ claimed that the power efficiency will also scale at the same pace with Moore’s law. Although the remarkable exponential scalability in integration still continues after five decades, Dennard’s law, which kept pace with Moore’s law for four decades, started to fail in 2004. The International Technology Roadmap for Semiconductors (ITRS)^3^ mentions a ‘red brick wall’ because there is no known technology solution below the 7-nm technology where complementary metal-oxide-semiconductor (CMOS) scaling is expected to stop (as already noted, CMOS power density scaling stopped in 2004). The two main causes for these limitations are the increases in leakage currents at such small scales (because the atoms do not scale with CMOS) and the limitations of electronics in interconnects (because of the skin effects and impedances of electronic interconnects). The arrival of chip multicore processors in 2006 is providing a temporary reprieve for these limitations by offering parallel processing capabilities in multicores without increasing the clock speed. An interesting new trend is the exponential increases in the number of cores in modern processors. In such parallel processing where interconnects are the bottlenecks, photonics provides parallelism and high capacity independently of distance. Hence, the hybrid integration of photonics and electronics can bring the best of both worlds where bosons (photons) and fermions (electrons) exhibit two sets of complementary traits. Photonic integrated circuits (PICs) take advantage of large bandwidth, low latency and low transmission loss, and electrical circuits exploit complicated signal processing. Table 1 lists commonly used photonic integration platforms and their attributes. Because the size of the electronics industry market is greater than four times that of the photonics counterpart, photonic integration platforms have always leveraged electronic integration counterparts such as CMOS for silicon and heterojunction bipolar transistor (HBTs) and high-electron mobility transistors for III–V platforms. Hence, it was not coincidental that the concept of co-integration of photonics and electronics emerged in the form of optoelectronic integrated circuits (OEICs)^4,5^ for III–V and CMOS-photonics for silicon^6^.

More importantly, both EICs and PICs have recently been seeking heterogeneous integration. Modern EICs often integrate heterogeneous circuits involving analog and digital or involving silicon CMOS and Ge bipolar transistor circuits. Likewise, photonic integration also benefits from integrating heterogeneous materials. Although silicon photonics is rapidly emerging as a viable and possibly ubiquitous photonic integration platform, silicon lacks optical gain, Pockels effect, and Faraday effect, which are useful for realizing lasers, phase modulators, and non-reciprocal devices. Hence, co-integration of silicon with III–V materials, electro-optical dielectrics, and magneto-optical materials can greatly enhance the functionalities of heterogeneously integrated microsystems.

As a substrate for an integration platform, the silicon substrate proves to be the most economical and scalable. The CMOS EIC industry is moving towards a 450-mm diameter silicon wafer platform with the ⩽14 nm gate CMOS technology, and 7 nm CMOS technology has recently been demonstrated. As a photonic device integration platform, silicon photonics is also gaining strong grounds. It is well known that the main reason behind the successful and practical development of multibillion-transistor circuits based on silicon CMOS is the availability of high-quality and dense passivation available from silicon’s natural oxide, SiO_2_. Interestingly, silicon photonics also benefits from the same high-quality silicon dioxide available for realizing high-contrast and low-loss Si/SiO_2_ waveguides exploiting a low-loss interface between silicon and SiO_2_. Owing to the availability of high-quality, large-scale, and low-cost silicon wafers together with a large number of CMOS foundries, silicon has recently emerged as a preferred integration platform.

Integration efforts in both EICs and PICs have thus far been active primarily in 2D. Because the number of transistors exceeded 10 billion per die, high-density integration has extended to 3D integration by stacking multiple layers of 2D EICs using through-silicon vias (TSVs)^7^. Three-dimensional EICs offered a number of performance enhancements over 2D EICs, primarily because of shorter electrical wiring requirements. They generally offer^8^ (a) lower power consumption due to a reduced number of repeaters and equalizers over shorter communication distances, (b) lower noise and jitter on shorter interconnects, and (c) higher packing density in 3D. In photonics, too, rapid advances in 2D photonic integrated circuits (PICs) have also motivated us to coin a term ‘photonic Moore’s Law’^9,10^. More recently, 3D photonic integration has emerged as very important steps towards bringing new functionality and a higher degrees of integration to microsystems^11–20^. For instance, space division multiplexing (SDM) based on 3D photonic integration overcomes limitations imposed on 2D photonics in handling the spatial degree of freedom and polarization dependence resulting from the fact that all waveguides must lie within the same 2D plane. Heterogeneous 2D and 3D photonic integration will bring diverse and complementary functions in a dense footprint. This paper overviews heterogeneous 2D and 3D photonic integration technologies and predicts their future directions.

## Monolithic versus hybrid heterogeneous integration in 2D

Heterogeneous integration can be achieved by either monolithic integration or hybrid integration. In particular, monolithic integration by hetero-epitaxy is attractive in that it can exploit large host substrates (for example, silicon wafers) and epitaxially grow III–V or other compound materials at a wafer scale at dimensions defined by the host substrate. For instance, InP lasers can be realized across 450-mm silicon wafers even when high-quality InP wafers are limited to ~75 mm diameters. However, the main challenge has been to overcome the lattice mismatch and to hetero-epitaxially grow low-defect materials. Epitaxial lateral overgrowth (ELO) technology has localized such defects within narrow apertures and grown reasonably high-quality crystals above dielectrics laterally seeded from the host materials through the apertures. Moreover, more recent work has exploited the 3D confinement of quantum dots to prevent the carriers from migrating to dislocations and demonstrated record performance on any lasers realized on silicon. In another recent work, the rapid-melt-growth (RMG) method has demonstrated high-quality germanium crystal growth from deposition of an amorphous germanium layer followed by rapid thermal annealing (melting), which is seeded by underlying silicon atoms reachable through nano-apertures formed similarly to those used for ELO. However, the RMG method is not as effective for III–V compound semiconductors on silicon because of twin formations in III–V materials. Overall, monolithic integration by heteroepitaxy remains challenging for achieving low-defect heterogeneous integration.

In contrast to monolithic integration, hybrid integration methods do not attempt material growth but utilize various bonding mechanisms between dissimilar (or similar) materials already grown on individual substrates. The bonding techniques^21^ known to microelectronics include (1) bonding without an interlayer, which includes (1a) anodic, (1b) direct, and (1c) low-temperature van der Waals bonding; (2) bonding with a metallic interlayer, which includes (2a) eutectic, (2b) thermos-compressive, and (2c) solder bonding; and (3) bonding with an insulating interlayer, which includes (3a) glass frit and (3b) adhesive bonding. For optoelectronics, the most popular bonding methods have been (1b) direct bonding (hydrophobic or hydrophilic) and (3b) adhesive bonding. Both direct bonding and adhesive bonding offer versatile heterogeneous integration between dissimilar materials, and the difference in thermal expansion coefficients and wafer sizes typically limit the choices of materials for wafer-scale integration. Direct bonding^22^ includes hydrophilic and hydrophobic bonding depending on the surface treatments prior to the bonding. In hydrophilic bonding, the hydroxyl of the atom yields water vapor after the bonding. Assuming silicon wafer to silicon wafer bonding, this process initially involves the following:

$$\;Si–OH+Si–OH=Si-O-Si+H_{2}O$$

The water vapor then binds with silicon to emit hydrogen gas: Si+2H_2_O=SiO_2_+2H_2_. In hydrophobic bonding, hydrogenated or fluorinated surfaces without oxides will bond to emit hydrogen gas: Si–H+Si–H=Si–Si +H_2_. Hence, in both cases, hydrogen gas can be trapped at the interfaces to cause defects, but this can be avoided by introducing vertical out-gas channels with silicon oxide to absorb the hydrogen molecules. Hydrophobic bonding requires higher bonding temperatures (>550 °C) but achieves stronger surface bonding energy (>2 J m^−2^) and intimate electrical bonding compared with hydrophilic bonding with a ~350 °C bonding temperature with an oxide layer at the bonding interface. Adhesive bonding using BenzoCycloButene (BCB)^23^, SU-8, or other interface layers has become very popular because of relatively relaxed requirements on the flatness of the bonding surfaces. Hydrophobic direct bonding has produced InP/InGaAsP edge-emitting lasers on GaAs substrates^22^, AlGaAs/GaAs edge-emitting-lasers on Si substrates^24^, and vertical cavity lasers with InP/InGaAsP active regions on GaAs/AlAs distributed Bragg reflectors^25^ with electrical currents flowing across the bonding interfaces. Hydrophilic direct bonding has produced AlInGaAs/InP-on-SOI hybrid lasers, modulators, and detectors achieving high-quality results with no obvious signs of additional defect-induced degradations, whereas the current flows are limited to the III–V regions without the ability to cross the bonding interface^26^.

## 3D Integration technologies

3D EICs include multiple layers of 2D EICs stacked and interconnected using TSVs^7^. Similarly, one type of 3D PICs have utilized orderly stacking of multilayer 2D photonic crystals to realize 3D photonic crystals^27^. Another type of 3D PIC utilized repeating of the combined processes of waveguide core layer deposition, lithography, etching, waveguide cladding layer deposition, and planarization (for example, chemical and mechanical polishing) to complete multilayer 3D PICs^28,29^ where interlayer coupling can be achieved by low-loss inverse taper waveguides^30^. Such 3D PICs can also be realized by wafer bonding of 2D PICs of similar^31^ or dissimilar^32^ materials while employing vertical couplers utilizing inverse taper waveguides for interlayer optical coupling. The interlayer optical coupling can also exploit photonic vias analogous to electrical TSVs, and a recent work^33^ has realized fabrication of photonic, electronic, and fluidic through-silicon vias in the same chip.

**Figure 1:**
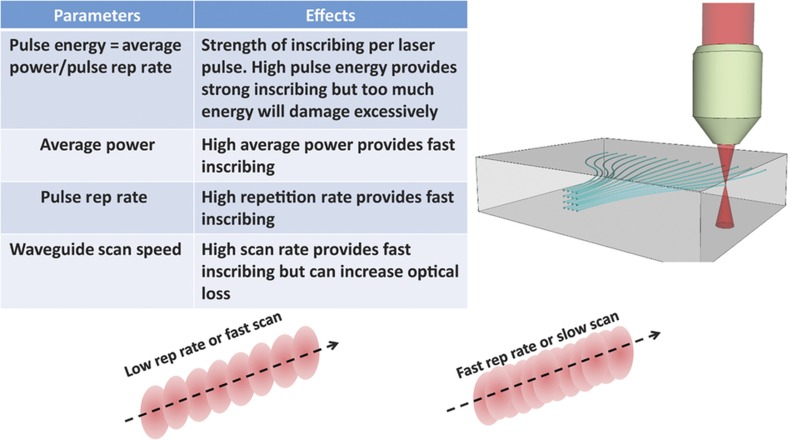
Schematic illustration of 3D photonic inscription where the waveguide is formed by increasing the local index of the material. The table shows how optical pulse energy, pulse repetition rate, and waveguide scan speed are adjusted to optimize the quality of the inscribed waveguide when the pulse width is held constant. Depending on the combination of laser repetition rate and mechanical scan rate, the material modification regions formed by each pulse will overlap by varying amounts. 3D, three dimensional.

**Figure 2:**
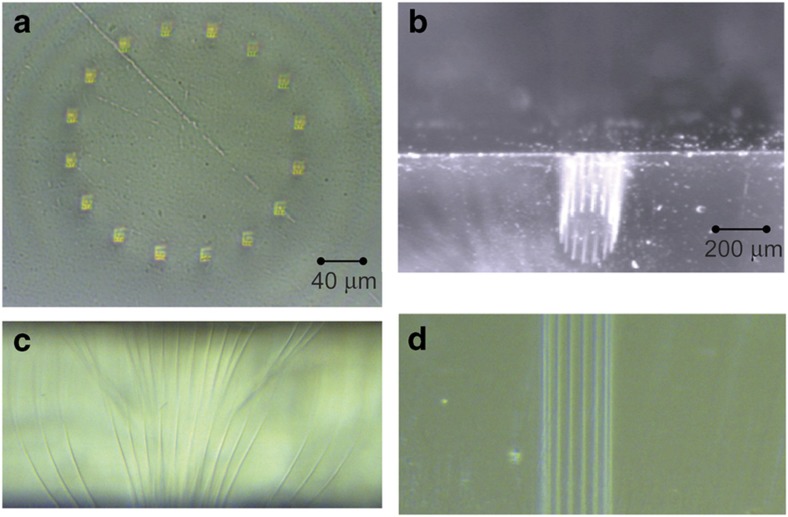
Microscopic images of a ULI-fabricated 3D waveguide fan-out device for orbital angular momentum applications^11,12,42–44^. (**a**) Facet image of the output showing the circular pattern of 16 waveguides. (**b**) View of **a** from above the top edge. (**c**) View of the interior where waveguides fan-out to a linear array. (**d**) View of waveguides near the output through the top surface. ULI, ultrafast laser inscription.

Perhaps, the most distinctive 3D PICs with no electronic counterpart are realized by 3D waveguide formation by ultrafast laser inscription (ULI). Direct laser writing of waveguides in dielectric material is an extremely powerful fabrication technique^34^. It utilizes the multi-photon nonlinear absorption of sub-bandgap photons to create permanent structural changes in a material with dimensions comparable to the writing laser’s wavelength (for example, ~1 μm^3^). The types of structural changes include refractive index and an increased susceptibility to chemical etching^35^. The induced modifications from a femtosecond train of optical pulses are strongly localized in three dimensions to the high-intensity region at the focus of a lens driven by a nonlinear absorption mechanism. This unique characteristic is what provides direct laser writing with its unique advantage over other waveguide fabrication techniques; the capability to freely form truly three-dimensional structures^36^. ULI has been widely demonstrated in many types of materials including amorphous glasses and crystals with measured propagation losses ⩽0.3 dB cm^−1^ (Refs. 37,38) in fused silica, similar to the 0.1 dB cm^−1^ propagation loss in 2D PICs (for example, Ge-doped silica on silicon)^37,38^. Furthermore, a recent demonstrations showed that high-quality three-dimensional waveguides are readily created with laser writing speeds on the order of 30 mm s^−1^ (Ref. 39). Finally, optical mode sizes can be adapted for a particular application by adjusting the geometry and composition of the inscribed waveguide by beam shaping of the inscribing laser or multiple scan techniques^40,41^. Figure 1 illustrates the ULI process that creates 3D waveguides within a bulk material where the waveguide core is formed by an increase in the local material index at the focus of the inscribing laser. For visible and near-infrared applications, fused silica and other glass materials are widely used for 3D waveguide inscription. Figure 2 shows an example of a 3D waveguide fan-out device that is fabricated by ULI. As detailed in the next section, the fan-out device can be butt-coupled to a 2D PIC (that is, planar lightwave circuit) for orbital angular momentum (OAM) applications^11,12,42–44^. In addition to silica, a variety of crystalline and non-crystalline materials can be used for 3D ULI waveguide applications. For instance, zinc selenide (ZnSe) is potentially a good 3D material because it is transparent from 0.5–20 μm and, although not as mature, low-loss waveguides in ZnSe^45–47^ and a solid-state Cr:ZnSe waveguide laser^48^ based on direct 3D laser writing have been demonstrated.

Similar laser inscribing methods applied to SU-8 or other ultraviolet-sensitive polymers can lead to the creation of ‘photonic wires’ through two-photon induced polymerization of negative-tone resist in the focus of a pulsed laser beam with a large numerical aperture^49^. Such polymerization using laser inscription creates 3D structures with relatively tight bending radii thanks to the high contrast between the polymer (core) and the air (cladding). Photonic wire bonding between various chips has been demonstrated using this technique^49^.

## Examples of 2D/3D integrated systems

### 2D/3D integrated OAM multiplexers

**Figure 3:**
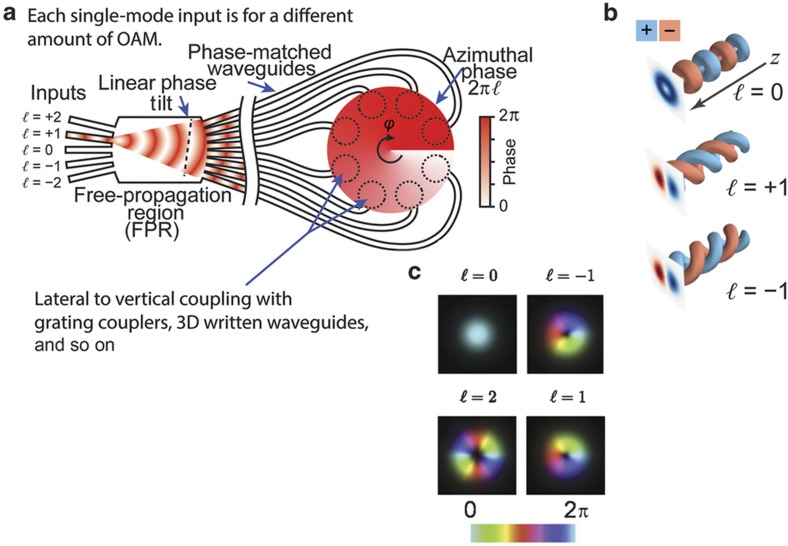
(**a**) Illustration showing how a beam encoded with an OAM state is sampled and demultiplexed by a circular arrangement of apertures, length-matched waveguides and a free-propagation region (FPR). (**b**,**c**) Visualization of the electric field of OAM beams.

Here we visit applications of heterogeneous integrated 2D and 3D systems. For instance, SDM based on 3D photonic integration overcomes limitations imposed on 2D photonics in handling the spatial degree of freedom and polarization dependence resulting from the fact that all waveguides must lie within the same 2D plane. Heterogeneous 2D and 3D photonic integration will bring diverse and complementary functions in a dense footprint. Figure 3a shows the integratable orbital angular momentum multiplexing device’s operating principle, which relies on converting linearly varying spatial phase to azimuthal variations (that is, exp(*ibℓx*)→exp(*iℓφ*), where *b* is the linear (*x*) to azimuthal (*φ*) scaling factor)^11,50^. To illustrate, Figure 3a shows a waveguide circuit in which each single mode input (that is, *ℓ*=−2, −1, 0, +1, +2) will create a wavefront in the free-propagation region (FPR) with a different linear tilt. The phase-matched waveguides after the FPR sample the tilted phase front and maintain the phase tilt to the output apertures. Because the apertures are arranged in a circular pattern, they create a beam (coming out of the page) with azimuthally varying phase having topological charge *ℓ*. If multiple inputs are illuminated, then those inputs are multiplexed onto collinear OAM beams with *ℓ*-numbers determined by the input position. By reciprocity, if an outside OAM beam illuminates the apertures, then the sampled light will be focused in the FPR to a waveguide corresponding to the beam’s *ℓ*-number (that is, an OAM demultiplexer). Working as a demux (that is, OAM state decoder), Figure 3b shows how a circular array of waveguide grating couplers are used to sample areas (dashed circles) of an incoming beam encoded with an OAM state (that is, *ℓ*=1) into a corresponding array of single-mode waveguides. Careful waveguide layout ensures that they have identical optical path lengths. Thus, at the input of the free-propagation region (FPR), the azimuthally varying phase of the OAM state is converted into a linear phase front with a tilt angle determined by the incoming beam’s topological charge, as indicated in Figure 3a. The circular placement of the array waveguides at the input of the FPR focuses the light, and the tilt of the linear phase front directs it to a corresponding output. The same PIC can function as a mux instead of demux when the light propagates in the opposite direction^42^.

**Figure 4:**
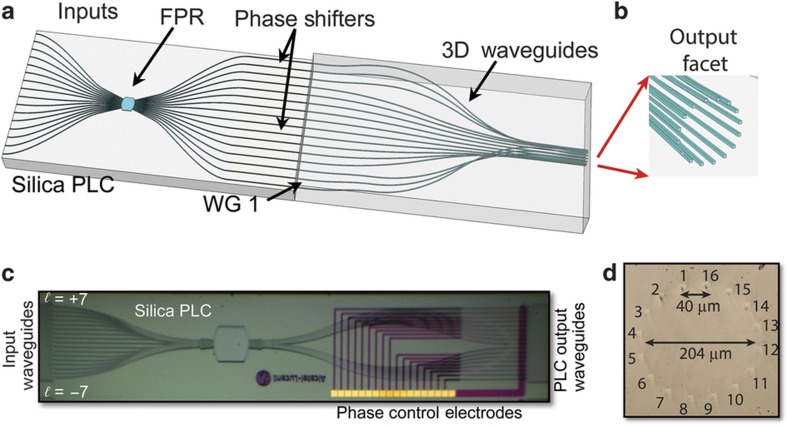
(**a**) Illustration of the silica PLC coupled to the 3D PIC. (**b**) 3D waveguide output facet detail. Estimated optical propagation loss is 0.2 dB cm^−1^. (**c**) Photo of fabricated silica PLC. (**d**) Photo of unpolished 3D PIC output face^11^. PIC, photonic integrated circuits.

We take advantage of the 3D capability to create the geometric transformation needed to convert linear phase tilt to azimuthal phase variation. Figure 4a shows how this concept is implemented using a silica PLC (to convert input position to a linear phase tilt) whose output is coupled to a 3D PIC for geometric transformation. Figure 4b presents a close view of the 3D PIC output face showing the circular arrangement. Figure 4c shows a photo of the fabricated PLC. The waveguides on the silica PLC have an index contrast Δ*n* of 2%. Electrical heaters on each output waveguide provide thermo-optic phase-error correction (PEC). This is used to phase match the waveguides between the FPR and the output face of the 3D PIC. Both the PLC and the input of the 3D PIC use a 127-μm waveguide pitch. The hybrid device (that is, PLC and 3D PIC) is ~30-mm long, and the waveguides on the output face form a 204-μm diameter circle with a center-to-center spacing of 40 μm (Figure 4d)^11^.

**Figure 5:**
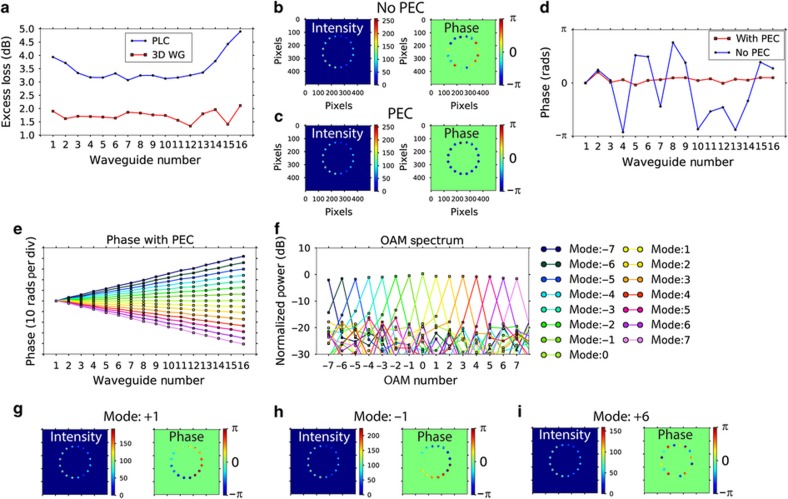
(**a**) Measured excess loss for the silica PLC and 3D waveguides. (**b**) Measured intensity and phase of the device’s output waveguides without PEC, (**c**) with PEC. (**d**) Measured average azimuthal phase at each output waveguide. (**e**) Unwrapped azimuthal phase for each OAM mode. (**f**) Calculated OAM mode purity. (**g**–**i**) The measured intensity and phase for three OAM modes (*ℓ*=+1, –1, +6)^11^. OAM, orbital-angular momentum; PEC, phase-error correction.

Figure 5 summarizes the optical characteristics of the combined 2D PIC: 3D PIC device of Figure 4a, including the waveguide losses (Figure 5a), whereas the optical path lengths are matched for all individual optical paths for the 16 apertures, phase error correction (PEC)^11^ is important for achieving very accurate relative optical phases between the apertures for realizing high-fidelity OAM multiplexer/demultiplexers (Figures 5b–d). Figures 5g–i shows examples of the measured intensity and phase of the OAM modes (for example, *ℓ*=+1, –1, +6), and Figure 5e summarizes the unwrapped phase plot versus waveguide output number for all OAM states. The result shows clearly that for each OAM state, the phase is the product of 2π and the OAM charge number *ℓ*. Figure 5f illustrates the crosstalk between the OAM states, which indicate less than −8 dB crosstalk. A higher number of waveguide apertures and more uniform power distribution after the FPR can reduce this crosstalk further.

### 3D multi-ring waveguides for OAM applications

**Figure 6:**
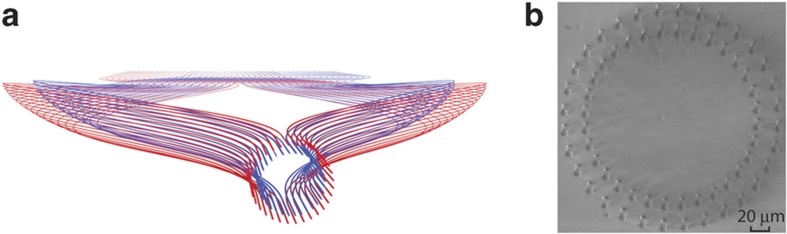
(**a**) Dual-ring 3D waveguide design utilizing the routing algorithm that maintains the same photonic path lengths, the minimum radius of curvature, and the minimum inter-waveguide distance, (**b**) a photograph of the fabricated multi-ring 3D waveguides (Corning Eagle 2000) observed at the facet. 3D, three dimensional.

Multi-ring 3D waveguide design and fabrication for the OAM application are important first step towards scalable SDM. Our group has developed a 3D waveguide design tool utilizing the routing algorithm that maintains the same photonic path lengths, the minimum radius of curvature, and the minimum inter-waveguide distance. Figure 6a shows the computer-aided-design, and Figure 6b is a photograph of the fabricated multi-ring 3D waveguides at the facet. In such cases, the multi-ring OAM PICs can possibly realize the generation of arbitrary spatial waveforms such as Laguerre–Gaussian modes by adding the radial control of the optical field to the azimuthal control achieved in individual OAM PICs of each ring.

### 3D waveguides for optical phased arrays

**Figure 7:**
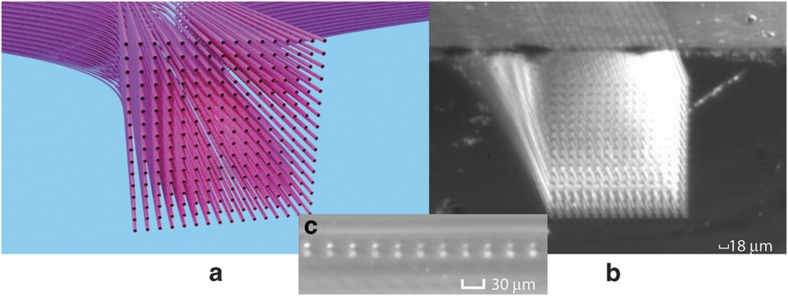
(**a**) 16×16 3D waveguide design utilizing a routing algorithm that maintains the same photonic path lengths, the minimum radius of curvature, and the minimum inter-waveguide distance, (**b**) a photograph of the fabricated 16×16 3D waveguides observed at the output facet. (**c**) Magnified micrograph of a portion of the 256 input waveguides. 3D, three dimensional.

**Figure 8:**
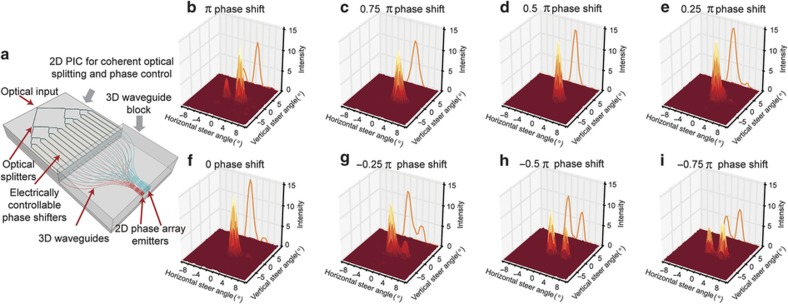
(**a**) Optical phase array beam steering module realized by heterogeneous integration of a 3D waveguide array similar to Figure 7b and a 2D PIC consisting of optical splitter waveguides and waveguide array phase shifters. (**b**–**i**) optical beam steering results obtained from phase tuning the array waveguides in the 2D PIC by π, 0.75π, 0.5π, 0.25π, 0, −0.25π, −0.5π, and −0.75π between the waveguides^51^. The optical waveguide propagation loss value of the 3D waveguide was ~0.2 dB cm^−1^. PIC, photonic integrated circuits; 2D, two dimensional; 3D, three dimensional

In addition to the spatial waveform shaping using multi-ring 3D PICs fabricated by ULI methods, the same ULI can form 3D PICs of arbitrary array shapes, including a rectangular array. Figure 7 shows the details of a 3D PIC that transforms a linear 1×256 input waveguide array into a 16×16 rectangular array of output waveguides using the ULI technique. Figure 7a shows a computer-aided design with details of the output array, Figure 7b presents a photograph of the output facet and Figure 7c presents a photograph of a portion of the input facet. As is the case with most dielectric waveguides, the index difference between the waveguide core and cladding determines the minimum bending radius due to radiation losses from the bent waveguide. Because the index difference for these ULI waveguides is ~0.5%, we chose a minimum bending radius of 20 mm, which showed negligible bending loss. Similar to the multi-ring 3D PICs, the rectangular PICs also utilized the 3D computer-aided design for precise optical path length matching of all 256 waveguides. Such 3D PICs are useful for non-mechanical beam steering^51^ and SDM MIMO optical communications and beamforming (for example, Hermite–Gaussian modes). Figure 8a shows an example of optical beam steering realized by heterogeneous integration of such a 3D waveguide array and a 2D PIC consisting of optical splitter waveguides and waveguide array phase shifters. Figures 8b–i illustrates optical beam steering results experimentally obtained from phase tuning the array waveguides in the 2D PIC by π, 0.75π, 0.5π, 0.25π, 0, −0.25π, −0.5π, and −0.75π between the waveguides^51^. Here PEC^11^ is conducted by phase tuning the array waveguides.

### Single-layer and multilayer 2D PICs for 2D/3D integration

**Figure 9:**
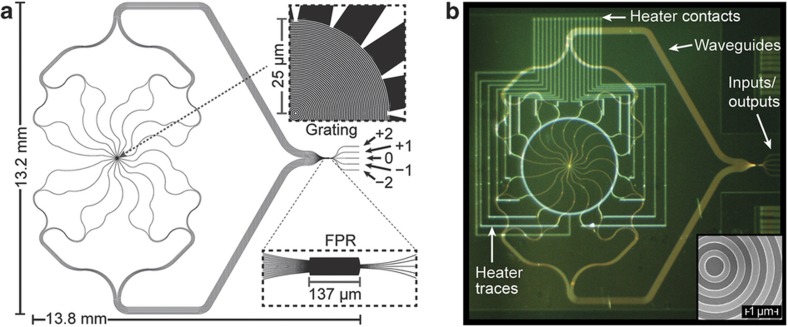
(**a**) Waveguide layout of silicon OAM device for multiplexing five OAM modes (ℓ=+2, +1, 0, −1, −2). (**b**) Fabricated silicon OAM device. The optical loss value was approximately 0.4 dB cm^−1^. The inset shows an SEM photo of the grating^42^. OAM, orbital-angular momentum; SEM, scanning electron microscope.

**Figure 10:**
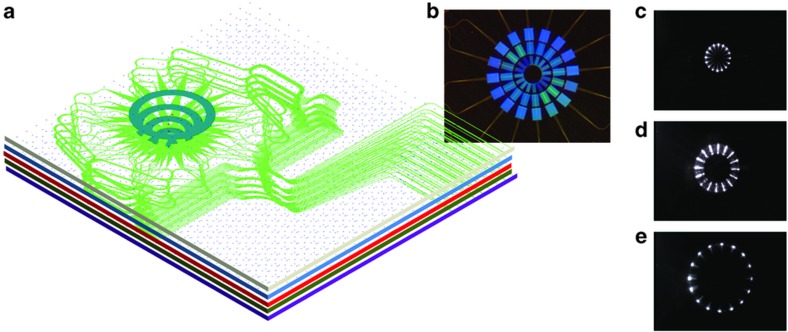
Stacking of multiple 2D layers for arbitrary waveform shaping. (**a**) Stacked device consisting of Layers 1–5. (**b**) Fabricated three-layer silicon nitride/silicon dioxide OAM device. Image of the three-layer silicon nitride/silicon dioxide OAM device when the (**c**) bottom (Layer 1), (**d**) middle (Layer 2), and (**e**) top (Layer 3) are illuminated. The optical waveguide propagation loss value was approximately 0.3 dB cm^−1^. OAM, orbital-angular momentum; 2D, two dimensional.

The OAM structures can also be fabricated by using a standard single-layer 2D PIC fabrication technique (for example, silicon photonic OAM mux/demux^42^), and multi-ring OAM structures can be realized by stacking and integrating multilayers of the single-ring OAM devices. Figure 9a shows^42^ the single-layer 2D silicon photonic integrated circuit layout, which is designed for a silicon-on-insulator (SOI) material platform, optimized for TE polarization, and uses a 1-μm-wide silicon rib waveguide (effective index of 3.27). The circular grating converts the vertically incident optical beam (azimuthal polarization) into a horizontally propagating beam. Depending on the OAM state of the input beam, the 16 guided modes will have a specific linear phase variation. Because the FPR is designed based on the Rowland circle principle, it focuses the 16 beams onto five waveguide outputs according to the linearly varying phase associated with the five different OAM states (labeled as *ℓ*=+2, +1, 0, −1, −2). We have extended a similar fabrication technique to the silicon nitride/silicon oxide waveguide system and utilized multilayer stacking of OAM device layers to realize multi-ring OAM PICs. Figure 10 shows stacking of multiple 2D layers for arbitrary waveform shaping, Figure 10a shows a stacked device consisting of Layers 1–5, and Figure 10b presents a fabricated three-layer silicon nitride/silicon dioxide OAM device. The images of the three-layer silicon nitride/silicon dioxide OAM device are also shown here when the (Figure 10c) bottom (Layer 1), (Figure 10d) middle (Layer 2), and (Figure 10e) top (Layer 3) are illuminated. When multiple layers are coherently excited, this device can create a mode profile that can excite eigenmodes of the multimode fibers. The main disadvantage of this stacked 2D waveguide approach compared with the laser-inscribed 3D waveguide approach is the poor support of polarization diversity^50^. The near-surface-normal emission using the gratings makes it difficult to support radial polarization, while reasonable throughput is achieved for azimuthal polarization.

**Figure 11:**
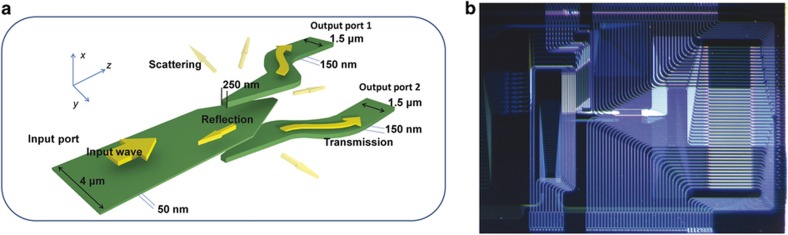
(**a**) Three-layer silicon nitride/silicon dioxide PIC with a 1×2 waveguide splitter^53^ and (**b**) a fabricated multilayer silicon nitride/silicon dioxide PIC^52^ designed to drive the multi-ring 3D PIC of Figure 6b. 3D PIC, three dimensional photonic integrated circuits.

In such multilayer 3D PICs, it is important to split or combine optical waves from one waveguide layer to another. Cross-layer couplers with extremely low losses at ~0.01 dB per coupler and low crosstalk of less than −42 dB per coupler have been designed and fabricated^52^ in silicon nitride/silicon dioxide waveguide PICs. Figure 11a shows a schematic of a three-layer silicon nitride/silicon dioxide PIC with a 1×2 waveguide splitter^53^ and Figure 11b presents a fabricated two-layer silicon nitride/silicon dioxide PIC^52^ designed to drive the multi-ring 3D PIC shown in Figure 6b.

The two types of 3D photonic integrated circuit fabrication technologies discussed here—(a) Multilayer 3D waveguides and (b) ultrafast laser inscription 3D waveguides—have their pros and cons. Table 2 summarizes their comparisons.

## Outlook and discussion

The continuing trend of exponential growth in data communications and processing will accelerate the pace of heterogeneous integration. In particular, we expect strong growth in the pace of 3D PIC development in addition to that of 2D PICs. Between the two main methods of realizing 3D PICs, the first method utilizing ULI is expected to see strong demand because of its capability to realize freely shaped waveguides of arbitrary contours and formations. Owing to the serial and sequential nature of its writing process, we expect expanded efforts to develop faster inscription recipes or to split laser beams for parallel and simultaneous writing of multiple PICs. On the other hand, the second 3D PIC fabrication method utilizing multilayer planar waveguides will also see strong demand supported, in part, by the availability of planar waveguide fabrication foundries. Heterogeneously integrated 2D and 3D PICs have shown promising results in SDM coherent optical communications and optical beam steering, and we believe that these results will bring new applications in spatial imaging, light detection and ranging (LIDAR), parallel optical links, and other emerging areas.

## Figures and Tables

**Table 1:** Various photonic integration platforms

PIC technology	Silicon PIC	GaAs PIC	InP PIC	Silica PIC
Leveraging technologies	Leverages Silicon CMOS industry	Leverages GaAs HBT industry	Leverages InP HBT industry	No electronics industry
Photonic–electronic integration	‘Silicon CMOS photonics’	‘GaAs OEIC’	‘InP OEIC’	Independent
Waveguide confinement	Strong confinement, Si/SiO_2_	Medium confinement GaAs/AlGaAs	Medium confinement InP/InGaAsP	Weak confinement GeO_2_, and so on doping
Typical MFD	<0.5 micron confinement	>2 micron confinement	>2 micron confinement	>5 micron confinement
Optical gain and optical modulation	No gain, no Pockel’s effect	Efficient gain, strong Pockel’s effect	Efficient gain, strong Pockel’s effect	No gain, no Pockel’s effect
Wafer size	⩾300 mm wafers	⩾200 mm wafers	⩾100 mm wafers	⩾200 mm wafers

Abbreviations: CMOS, complementary metal-oxide-semiconductor; HBT, heterojunction bipolar transistor; PIC, photonic integrated circuits.

**Table 2:** Comparisons of the two types of 3D photonic integrated circuit fabrication technologies

3D PIC technology	Multilayer 3D waveguides	Ultrafast laser inscription 3D waveguides
Pros	Precise lithographic fabrication; wafer-scale process; based on mature technologies; Dense waveguide layout; High index contrast	Single-step fabrication process; low-loss waveguides Flexible integration with 2D PICs
Cons	Multiple step fabrication process; stresses induced by multiple layers; Non-uniform performance	Low index contrast; Large bending radius; difficult to scale fabrication

Abbreviation: 3D, three dimensional; 2D PIC, two-dimensional photonic integrated circuits.
